# Obsessive-Compulsive Disorders and Functional Urinary Disorders: A Fortuitous Association?

**DOI:** 10.3390/bs11060089

**Published:** 2021-06-17

**Authors:** Qin Xiang Ng, Yu Liang Lim, Wayren Loke, Wee Song Yeo, Kuan Tsee Chee

**Affiliations:** 1 MOH Holdings Pte Ltd., 1 Maritime Square, Singapore 099253, Singapore; yulianglim95@gmail.com (Y.L.L.); wayren.loke@mohh.com.sg (W.L.); 2 Mount Elizabeth Hospital, 3 Mount Elizabeth, Singapore 228510, Singapore; corneliuslionel@gmail.com; 3 Department of General and Community Psychiatry, Institute of Mental Health, 10 Buangkok View, Buangkok Green, Medical Park, Singapore 539747, Singapore; kuan_tsee_chee@imh.com.sg

**Keywords:** urinary incontinence, functional, enuresis, obsessive-compulsive, rituals

## Abstract

Although psychological factors are known to affect bladder and bowel control, the occurrence of functional urinary disorders in patients with psychiatric disorders has not been well-studied or described. A higher prevalence of functional lower urinary tract disorders have also been reported amongst patients with obsessive-compulsive (OC) disorders. A systematic literature search of PubMed, EMBASE, OVID Medline, PsycINFO, Clinical Trials Register of the Cochrane Collaboration Depression, Anxiety and Neurosis Group (CCDANTR), Clinicaltrials.gov and Google Scholar databases found five observational studies on the topic. Unfortunately, as only one study had a (healthy) control group, a meta-analytic approach was not possible. Overall, patients with OC symptoms appeared to have increased occurrence of functional urinary symptoms, e.g., overactive bladder, increase in urgency, frequency, incontinence and enuresis. This was even more common amongst patients with Pediatric Autoimmune Neuropsychiatric Disorder Associated with Streptococcal Infections (PANDAS) or Pediatric Acute-onset Neuropsychiatric Syndrome (PANS) as opposed to patients with OCD alone. Several biological and behavioural mechanisms and treatment approaches were discussed. However, as the current evidence base was significantly limited and had moderate to serious risk of bias, no strong inferences could be drawn. Further well-designed cohort studies are necessary to better elucidate the observed associations and their management.

## 1. Introduction

Urinary incontinence is a common and often impairing and socially embarrassing occurrence [1]. It is increasingly recognized as a potential symptom in patients with psychiatric disorders [2]. Although psychological factors (including depression and anxiety) are known to affect bladder and bowel control [3], the occurrence of functional urinary disorders in patients with psychiatric disorders has not been well-studied or described. 

Functional lower urinary tract disorders comprise a diverse group of disorders, for which there is no apparent neurological disease and the structural aspect remains unknown [4]. They are frequently comorbid in children with attentional and obsessive-compulsive (OC) disorders [5,6,7]. Some case reports have suggested that urination could be a form of compulsion [8,9]; cognitive behavioural therapy (CBT) has long been applied in the treatment of functional urinary disorders [10]. It has also been hypothesized that these conditions may at least share some common neuropharmacological pathways involving stress-related peptide corticotropin-releasing factor (CRF) and CRF-related peptides [11], and the treatment of one may improve the other. 

It is important for clinicians to promptly recognize and manage these conditions as the experience of incontinence in children and adolescents can perpetuate poor mental health and increase one’s risk of social isolation, bullying and self-esteem issues [12]. However, understanding of the potential Psyche–Soma relationship and its interactions remains underdeveloped.

To the best of our knowledge, no systematic review or meta-analysis has been done hitherto to investigate the extent of the association between OC disorders and functional urinary disorders in either adults or children. It was hypothesized that patients with OC symptoms would also have an increased occurrence of functional urinary symptoms. This study aimed to investigate this a priori hypothesis, summarize current evidence and suggest areas for future research.

## 2. Methods

A systematic literature search was performed in accordance with the latest Preferred Reporting Items for Systematic Reviews and Meta-Analyses (PRISMA) guidelines [13]. By using the following combinations of broad Major Exploded Subject Headings (MesH) terms or text words [urinary OR incontinence OR enuresis OR overactive OR bladder] AND [obsess * OR compuls * OR OC OR OCD OR yale-brown], a comprehensive search of PubMed, EMBASE, OVID Medline, PsycINFO, Clinical Trials Register of the Cochrane Collaboration Depression, Anxiety and Neurosis Group (CCDANTR), Clinicaltrials.gov and Google Scholar databases yielded 2359 papers published in English between 1 January 1988 and 1 April 2021. Attempts were made to search grey literature using the Google search engine. Title/abstract screenings were performed independently by three study investigators (Q.X.N., Y.L.L. and W.L.) to identify articles of interest. All retrieved publications were manually reviewed and also checked for references of interest. 

The inclusion criteria for this review were: (1) published clinical study, (2) study population with a diagnosis of OCD, and (3) reported occurrence or prevalence of functional urinary disorders (e.g., overactive bladder, functional urinary incontinence, etc.). Any disagreement on inclusion was resolved by consensus. Exclusion criteria included case reports, case series, conference abstracts and proceedings, which were not accepted for this systematic review.

Data were extracted using a standardized electronic form by one study investigator (Q.X.N.) and cross-checked by a second investigator (Y.L.L.) for accuracy. The quality and risk of bias of the studies were also assessed with the ROBINS-I (Risk of Bias in Non-randomized Studies-of Interventions) tool [14], graded based on consensus of three study investigators (Q.X.N., Y.L.L. and W.L.).

## 3. Results

**Figure 1:**
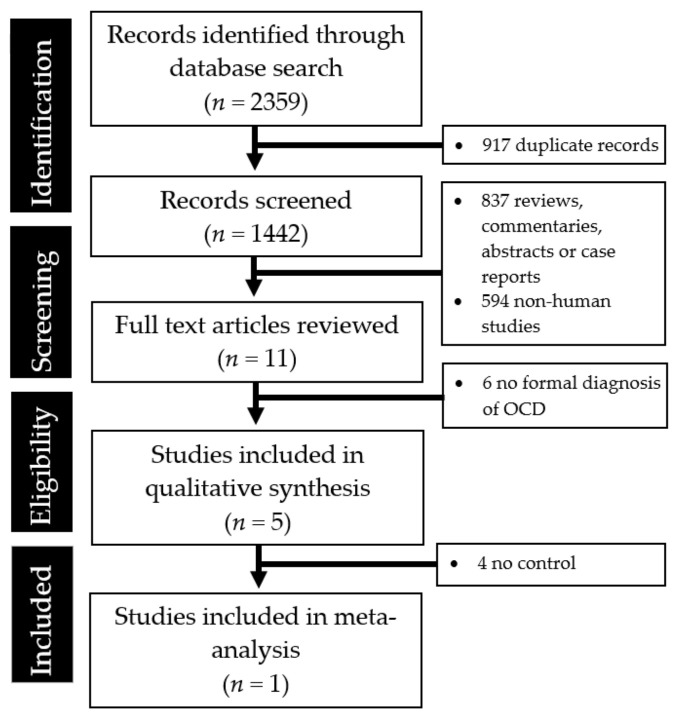
PRISMA flow diagram summarizing the studies identified during the literature search and abstraction process.

The abstraction process (and reasons for exclusion) is summarized in Figure 1. 

A total of five observational studies were systematically reviewed. The details of the studies, including the study demographics, are summarized in Table 1. Unfortunately, as only one study had a (healthy) control group, a meta-analytic approach was not possible. For the same reasons, a sensitivity analysis was not performed. 

In terms of the quality of the available studies, most were observational in nature, had relatively small sample sizes and overall moderate to serious risk of bias, due to the lack of control for potential confounding factors and selection bias, as seen in Table 2.

## 4. Discussion

Overall, patients with OC symptoms appeared to have increased occurrence of functional urinary symptoms, e.g., an increase in urgency, frequency, incontinence and enuresis. This appeared to be even more common amongst patients with PANDAS/PANS as opposed to patients with OCD alone [17,18,19].

There are many causes of urinary incontinence and they can be broadly classified as stress incontinence (related to weakness of the pelvic floor support and increases in intraabdominal pressure), urge incontinence (believed to be due to detrusor overactivity), overflow incontinence, mixed incontinence, or functional incontinence (thought to be caused by non-genitourinary factors, such as cognitive or physical impairments, affecting one’s ability to void independently) [20]. Psychogenic polydipsia is more common in patients with schizophrenia than patients with OCD [21]. It is thought that some patients with OCD may have delayed voiding of the bladder because of certain contamination fears or OC rituals, resulting in functional incontinence. To remedy this, adherence to a strict bowel program and timed voiding regimen may be helpful [16]. Clinicians may schedule for them to urinate every two to four hours. 

There is also a proposed subtype of OCD patients who have urinary obsessions and they may report predominant fear of urinary incontinence and urinary frequency without organic etiology [22]. These patients appear to respond to antidepressants. The treatment of OCD may be very complex and refractory in some cases and could require add-on atypical antipsychotic medication and psychotherapies [23].

Another potential contributory factor include the psychotropic medications themselves. The lower urinary tract is influenced by various neurotransmitter pathways. In literature, there have been several case reports of urinary incontinence associated with the use of selective serotonin reuptake inhibitor (SSRI) antidepressants [24] and antipsychotics over a broad range of doses [25]. In terms of biological mechanisms, SSRIs may have agonist effects on 5-HT4 receptors, which are also found in the urinary tract and could evoke responses in the bladder and urethra [26]. In isolated detrusor muscle strips from guinea pigs, 5-HT4 receptors were found to potentiate purinergic transmission and thus initiate the voiding reflex [27]. A retrospective chart analysis of 13,531 first-time users of an SSRI also found that the adjusted relative risk of urinary incontinence with SSRI exposure (compared to non-exposure) was 1.61 (with a 95% confidence interval of 1.42 to 1.82) [28]. This risk was higher amongst females, the elderly and patients prescribed sertraline compared to the other SSRI antidepressants. As for both typical and atypical antipsychotics, they may affect detrusor overactivity and reduce bladder compliance [29]. However, the mechanisms remain unclear and individual susceptibility may differ.

In addition to the serotonergic and antipsychotic drugs, clomipramine could also induce urinary symptoms in patients with OCD [30], as can happen with benzodiazepines and other drugs [31].

Unfortunately, the current evidence base was significantly limited and a meta-analytic approach was not possible. The studies were also observational in nature, had relatively small sample sizes and overall moderate to serious risk of bias. This also meant that we were unable to prove causation and the observations could indeed be fortuitous. There was also the possibility of ascertainment bias with case collection studies. The conclusions must thus be interpreted in light of these shortcomings. 

Further well-designed cohort studies on the subject are necessary to clarify these observed associations and their management.

## 5. Conclusions

In conclusion, our systematic review found five observational studies, which all showed increased occurrence of functional urinary symptoms in patients with OC symptoms. In clinical practice, it is known that patients with OCD may manifest with various symptoms, however, it remains unclear if the observed functional urinary symptoms are fortuitous, biological or even behavioral in nature. Nonetheless, as urinary incontinence can be particularly distressing and socially embarrassing for the individual, this should be sensitively elicited in the clinical history and approach to these patients. The importance of a good history and a holistic understanding of the patient’s psychiatric phenomenology cannot be overstated. There are still significant gaps in our present understanding of this Psyche–Soma relationship, which could be the result of biological feedback, medications or other neural pathways. Further well-designed cohort studies on the subject would help shed light on these associations and their management.

## Figures and Tables

**Table 1:** Clinical studies reporting the occurrence of functional urinary disorders in patients with OC symptoms.

Author, Year	Country of Origin	Study Design and Study Population	Key Findings
Ahn et al., 2016 [15]	Korea	Case-control; *n* = 114 (57 women with OAB and 57 healthy female controls); Average age 50.65 ± 14.72 years	- Significant association between OAB and OC symptoms (OR 5.47 (95% CI: 1.99 to 15.04, *p* = 0.001) compared to control group - Consider screening for OCD and appropriate psychiatric referral for female patients with OAB
Arlen et al., 2014 [16]	United States	Retrospective chart review; *n* = 20 (2 boys and 18 girls); Average age 6.9 (range: 4 to 12 years)	- All children presented with the sensation of urinary leakage despite being completely dry and had normal physical examination and investigations. - Most children (75%) also experienced urinary urgency and half (50%) had increased urinary frequency (voiding more than eight times a day). - 70% (*n* = 14) had a prior diagnosis of OCD or OCD traits, as reported by their parents.
Bernstein et al., 2010 [17]	United States	Case-control; *n* = 39 (24 boys and 15 girls); Average age 10.66 years	- Urinary symptoms (including increase in urgency, frequency and enuresis) were much more common in children with PANDAS as opposed to non-PANDAS OCD. - There were no significant differences between the two groups of children in terms of demographics or OCD severity rating.
Jaspers-Fayer et al., 2017 [18]	Canada	Prospective cohort; *n* = 136 (54% males); Age range 6 to 19 years	- Parents of children with PANDAS/PANS were more likely than parents of children with OCD to report a history of urinary incontinence (57%, *n* = 4 out of 7 vs. 18%, *n* = 17 out of 92, *p* = 0.035).
Murphy & Pichichero, 2002 [19]	United States	Prospective case identification and followup; *n* = 12 (7 boys and 5 girls); Average age 5 years (range: 5 years 4 months to 10 years 11 months)	- Of the 12 children with PANDAS, 7 (58%) had compulsive daytime urinary urgency, frequency, and wiping, without fever, dysuria, nighttime symptoms or evidence of UTI.

Abbreviations: CI, confidence intervals; OAB, overactive bladder; OCD, obsessive-compulsive disorder; OR, odds ratio; PANDAS, pediatric autoimmune neuropsychiatric disorders associated with streptococcal infections; PANS, pediatric acute-onset neuropsychiatric syndrome; UTI, urinary tract infection.

**Table 2:** Risk of bias assessment with ROBINS-I tool.

Study	Confounding	Selection	Measurement of Intervention	Missing Data	Measurement of Outcomes	Reported Result	Overall
Ahn et al., 2016 [15]	Serious	Serious	N/A	?	Moderate	Moderate	Serious
Arlen et al., 2014 [16]	Serious	Serious	N/A	?	Serious	Serious	Serious
Bernstein et al., 2010 [17]	Serious	Serious	N/A	?	Moderate	Moderate	Serious
Jaspers-Fayer et al., 2017 [18]	Moderate	Serious	N/A	?	Moderate	Moderate	Serious
Murphy & Pichichero, 2002 [19]	Serious	Serious	N/A	Moderate	Serious	Moderate	Serious

Abbreviations: ?, insufficient information to determine risk of bias; N/A, not applicable.

## Data Availability

The datasets generated during and/or analysed during the current study are available from the corresponding author on reasonable request.
